# Complete genome sequence of *Methylocystis echinoides* strain RIM isolated from soil

**DOI:** 10.1128/mra.01155-24

**Published:** 2025-02-25

**Authors:** Ahmed M. Abdel-Hamid, Taylor Putman, Megan Furukawa, William Palivos, Kimberly K. O. Walden, Christopher J. Fields, Patrick Schimmel, Roderick I. Mackie, Isaac Cann

**Affiliations:** 1 Carl R. Woese Institute for Genomic Biology, University of Illinois Urbana-Champaign, Urbana, Illinois, USA; 2 Energy & Biosciences Institute, University of Illinois Urbana-Champaign, Urbana, Illinois, USA; 3 Department of Animal Sciences, University of Illinois Urbana-Champaign, Urbana, Illinois, USA; 4 Roy J. Carver Biotechnology Center, University of Illinois Urbana-Champaign, Urbana, Illinois, USA; 5 Division of Nutritional Sciences, University of Illinois Urbana-Champaign, Urbana, Illinois, USA; 6 Department of Microbiology, University of Illinois Urbana-Champaign, Urbana, Illinois, USA; 7 Center for East Asian and Pacific Studies, University of Illinois Urbana-Champaign, Urbana, Illinois, USA; University of Maryland School of Medicine, Baltimore, Maryland, USA

**Keywords:** *Methylocystis*, methanotrophs

## Abstract

We report here the complete genome sequence of a type II methanotrophic bacterium, *Methylocystis echinoides* strain RIM, isolated from the soil surface at Urbana, Illinois. This genome was obtained via HiFi PacBio Sequel II sequencing.

## ANNOUNCEMENT

Methane, the primary component of natural gas, is a potent greenhouse gas that affects earth temperature (1). Methane also provides a low-cost carbon feedstock that can be converted by methanotrophic bacteria to liquid biofuel and other value-added products (2). The oxidation of methane by methanotrophs contributes to the mitigation of global climate change (3). *Methylocystis echinoides* is a type II methane-oxidizing, strictly aerobic, gram-negative bacterium (4). *Methylocystis* species possess particulate methane monooxygenase and utilize the serine pathway for carbon assimilation (5). Soil samples were collected from the edge of a lake located near Japan House, Urbana, Illinois, USA (GPS coordinates 40.092956–88.217487). The growth of methanotrophs was enriched on nitrate mineral salt (NMS) medium containing methane (1:2, Methane:Air) as the sole carbon source. Enrichment was performed for 3 weeks at 30°C. The enriched culture was serially diluted, and single colonies were then obtained by spread plating on NMS agar plates and incubation at 30°C in anaerobic jars supplemented with 50% methane. A single colony was picked and further purified by streaking three times on an NMS agar plate. A pure colony was grown in an NMS culture medium and genomic DNA was extracted using Qiagen genomic-tip 20 /G (Qiagen, MD, USA). DNA concentration and integrity were assessed by using a ND-1000 NanoDrop spectrophotometer and resolving on a 1% agarose gel. The bacterium was identified by amplifying the full-length 16S rRNA gene by Phusion DNA polymerase (NEB, MA, USA) using the 27F and 1492R primers followed by Sanger sequencing at the Roy J. Carver Biotechnology Center at University of Illinois Urbana-Champaign. The 16S rRNA gene sequence showed 99.5% identity with 99% query cover to *Methylocystis echinoides* strain IMET 10491 (accession number NR_025544.1). The genomic DNA of *Methylocystis echinoides* RIM strain was sheared with a Megaruptor 3 to an average fragment length of 13 kb. The sheared DNA fragments were barcoded and converted to a library with the SMRTBell Express Template Prep kit 2.0 (PacBio, CA, USA). The library was quantitated with Qubit and run on a Femto Pulse (Agilent, CA) to confirm the presence of DNA fragments of the expected size. The library was sequenced on 1 SMRT cell 8M on a PacBio Sequel IIe using Binding Kit 2.0 with the V4 primer with the CCS sequencing mode and a 30hs movie time. CCS analysis and adaptor trimming were done using SMRTLink v10.0 using the following parameters: ccs --min-passes 3, --min-rq 0.99, lima—ccs --same --split-bam-named. The PacBio library yielded 1.762 Gb of HiFi reads, which were filtered with seqkit version 0.12.1 (6) to remove reads less than 1 kb and greater than 50 kb. Filtered reads were then down-sampled to approximately 50× genome coverage with Filtlong v0.2.1 (7). Down-sampled reads were assembled with Flye assembly version 2.8.2 (8) using the estimated genome size, the option "--hifi-error 0.003," and the option “--plasmids” to rescue short plasmids. Circlator version 1.5.1 (9) then circularized and set the starting point of the Flye assemblies, using the options "--merge_min_id 85," "--merge_breaklen 1000," and "--assembler canu." Following the assembly process, the resulting contigs amounted to a cumulative size of 4.35 Mb. This assembly achieved coverage of approximately 405×, calculated by dividing the total HiFi bases by the contig size. The assembly unveiled four circular contigs, with distinct characteristics. The largest contig (3.7 Mb) was predicted to represent the chromosome of *M. echinoides* strain RIM. Additionally, a 299,383 bp contig, a 166,303 bp contig, and a 137,443 bp contig were predicted to be circular plasmids. The genome assembly was annotated using the NCBI Prokaryotic Genome Annotation Pipeline (PGAP) (10). Detailed information for the genome is provided in Table 1.

**TABLE 1:** Genome characteristics of *M. echinoides* strain RIM isolated from soil

Characteristic	Data
Whole-genome characteristics	
BioSample accession no.	SAMN37666069
Total no. of reads	151,210
Read N50	13,244 bp
SRA accession no.	SRX22177046
Genome size (no. of bp)	4,352,665
Genome coverage	405 x
GC Content (%)	64
Total no. of genes	4,301
No. of chromosomes + plasmids	1 + 3
Chromosome	
GenBank accession no.	CP156084
Chromosome topology	Circular
Chromosome size (bp)	3,749,536
GC Content (%)	64.5
Total no. of chromosomal genes	3,706
No. of protein-coding genes	3,627
No. of rRNAs	6
No. of tRNAs	50
No. of other RNAs	4
No. of pseudogenes	19
Plasmids:	
Plasmid name	pME_1
GenBank accession no.	CP156085
Size (bp)	299,383
GC content (%)	60.5
No. of genes	306
No. of protein-coding genes	265
Plasmid name	pME_2
GenBank accession no.	CP156086
Size (bp)	166,303
GC content (%)	65.5
No. of genes	160
No. of protein-coding genes	158
Plasmid name	pME_3
GenBank accession no.	CP156087
Size (bp)	137,443
GC content (%)	60
No. of genes	129
No. of protein-coding genes	124

## Data Availability

This sequence has been deposited in the GenBank database under BioProject accession number PRJNA1023607
